# The relevance of the social networking media in Ebola virus disease prevention and control in Southwestern Nigeria

**DOI:** 10.11694/pamj.supp.2015.22.1.6165

**Published:** 2015-10-10

**Authors:** Wasiu Olalekan Adebimpe, Damilare Hakeem Adeyemi, Ayodeji Faremi, John Olujide Ojo, Adeola Ebun Efuntoye

**Affiliations:** 1 Department of Community Medicine, College of Health Sciences Osun State University Osogbo, PMB 4494 Osogbo, Nigeria; 2 Physiology Department, University of Ibadan, Ibadan, Nigeria; 3 Osun State Hospitals Management Board, Osogbo, Nigeria; 4 Department of Community Medicine, Federal Teaching Hospital Ido Ekiti Nigeria; 5 Family Health International 360, Abuja, Nigeria

**Keywords:** Ebola Virus Disease, social media, misconceptions

## Abstract

**Introduction:**

The Social Media (SM) is fast becoming a huge avenue to create and spread health awareness amongst youths. Meanwhile news surrounding the on-going Ebola Virus Disease (EVD) outbreak in West Africa are frightening. This study assessed the relevance of the social networking media in spreading awareness about EVD prevention and control in Southwestern Nigeria.

**Methods:**

Descriptive cross sectional study among 400 youths selected using multistage sampling method. Research instruments used were semi structured self administered questionnaires that were analyzed using the SPSS software version 17.0.

**Results:**

Three hundred and eighty (95.0%) of respondents were members of a social network. Several misconceptions trailed the spread of information about EVD. Though only 21(7.3%) bothered to ascertain the reliability of such information before use, 332 (83.0%) believed that SM can also be used to dispel rumours on health information, 337(84.3%) said they would like the use of SM regulated, while 206 (51.6%) still believed that SM is effective in disseminating health information among youths. Only 79.4% had good knowledge of EVD, and 30.7% of respondents had misinformation about EVD. Determinants of good level of knowledge about EVD through SM contacts include being a male, having SM as the first source of information on EVD and believing that SM could assist to disseminate and improve health information.

**Conclusion:**

Misconceptions spread through the SM fuelled the ongoing EVD outbreak in West Africa. There is a need for the concerned authority to create awareness through SM contacts as well as regulate its use.

## Introduction

The second half of the year 2014 witnessed a large epidemic of EVD in the West African sub-region, resulting in several thousands of cases and hundreds of deaths. Ebola is one of the filo-viruses and among the most virulent pathogens of humans, causing severe hemorrhagic fever [1, 2], giving case fatality rates of between 80 to 90 percent during epidemics [3]. News going round about EVD are frightening, and it has fostered suspicion, anxiety, myths and misconceptions in communities and within health care settings, stressing the need to spread awareness of EVD among all categories of people.

The SM is fast becoming a huge medium to spread health awareness amongst the people with the advent of the Mobile telecommunication technology. On a daily basis, about five to six hundred million people visits social networking sites like Facebook and Twitter, thus creating an online community that drives the creation of awareness in many different ways and for multiple purposes [4].

Users of SM networking sites could repackage the information they received or find for others, create a forum for knowledge discovery and discussions and receiving feedbacks [5, 6]. However, the veracity of information spread through this medium may require verification to ensure a reasonably level of validity. Dispelling these fears, myths and misconceptions is perhaps the biggest challenge in tackling Ebola Virus Disease outbreak. This study assessed the role of the social networking media in spreading awareness about EVD prevention and control in Southwestern Nigeria.

## Methods

**Study area:** Osun State University is one of the two higher education level institutions in Osogbo, the capital of Osun state. The school runs a multi-campus system. Nigeria was certified EVD free by the WHO in September 2014 after about six months of EVD outbreak that ravaged the country. Education institutions had the resumption of academic activities of their students from holidays postponed to allow for better control of the disease. In Osun State, mobile phones are accessible to an average youth.

**Study design:** the study was a descriptive cross sectional study of the role of the social media on the spread of information about EVD among undergraduates of Osun State University Oshogbo.

**Sample size estimation:** using Leslie Fischer's formular for calculation of sample size for population above 10,000 and prevalence figure of use of mobile network of 0.9, a sample size of 238 was calculated, and this was increased to 400 for better representation as well as to account for non response.

**Sampling methods:** a multistage sampling method was adopted in sample selection. In the second one, two out of six Colleges were selected using simple random sampling employing simple balloting. In stage 2, three Departments per College were selected using simple random employing simple balloting. Questionnaires were proportionately allocated to the Colleges and the twelve departments selected.

In stage three, stratified sampling method was used in selecting the classes using their level of education as the stratifying factor. One level or class per department was randomly selected. In stage four, respondents were recruited into the study using a systematic sampling of one in three according to that day's sitting arrangement in the lecture hall. Questionnaires allocated to the classes were distributed until they got exhausted. In the class where questionnaires were not exhausted, another class was chosen using simple random sampling and respondents recruited in the same way.

**Research instruments:** include semi structured self administered and pre tested questionnaires Pre testing was done among 20 students of a polytechnic in nearby Oyo State, and the responses used in questionnaire modification. Questionnaires were divided into 2 parts namely socio demographic characteristics and the role of the social media in spreading awareness of EVD and prevention and control.

**Ethical approval:** to conduct this study was obtained from LAUTECH Teaching Hospital health ethics committee. Further permission was obtained from Heads of Departments of students who participated in the study. A written informed consent was also obtained from each student participant.

**Data management:** questionnaires were manually sorted out followed by data cl**e**aning. Data were entered into the SPSS software version 17.0 after ensuring validity checks through double entry, random checks and looking for outlier variables. Frequency tables and charts were generated including calculation of relevant summary indices. Bi variate analysis of some variables in relation to the relevance of SM was carried out in addition to some related binary logistic regression. P value was considered statistically significant at values <0.05 for all inferential statistics.

## Results

The age group 16 to 20 years constitutes the highest 194(48.5%) age group among respondents; mean age of respondents was 21.2 (+2.4) years. Two hundred and twenty six of respondents were females, only 24(6.0%) were married, 289(72.3%) were Christians while the classes were evenly distributed among respondents (Table 1). Table 2 showed that 380(95.0%) of respondents were members of a social network, commonly Facebook and Twither. The social network media was the first source of information on EVD among 287(71.8%) of respondents.

**Table 1:** Personal data of respondents

Variable	F	%
**Mean age = 21.2** ± **2.4 years Age group 9 in years)**		
16-20	194	48.5
21-24	141	35.3
25-29	54	13.5
30-34	11	2.7
**Sex**		
Male	173	43.3
Female	226	56.5
**Marital status**		
Ever married	24	6.0
Never married	376	94.0
**Religion**		
Christianity	289	72.3
Islam	99	24.8
Traditional	10	2.5
Others	2	0.5
**Class**		
I	100	25.0
II	100	25.0
III	100	25.0
IV	100	25.0

**Table 2:** Common misconceptions and relevance of social media in improving EVD awareness

Variable (n = 400)	F	%
Respondent is a member of a social network	380	95.0
**Common social networks (multiple responses) n = 380**		
Facebook	355	93.4
Twitther	87	22.9
Linkdeth	20	5.3
Others	80	21.1
Social media was the first source of information about EVD	287	71.8
That it is a new disease	207	51.8
That it kills very fast	292	73.0
That it is incurable	316	79.0
**Common misconceptions (n = 287)**		
Can be treated with antibiotics	51	17.8
Prevented by taking daily hot water bath with salt	214	74.6
Prevented by taking bitter cola	237	82.6
Prevented by taking miracle cola	82	28.6
Prevented by frequent rubbing of the body with Aloverae soap and cream	40	13.9
Prevented by drinking plenty of condensed milk	23	8.0
Curable by taking appreciable quantity of onions	46	16.0
Preventable by not shaking hands with friends and colleagues	125	43.6
Did you beliefs in all these received messages at first exposure) n = 287	218	76.0
Did you forward these messages to others	150	52.3
Did you get a feedback from anyone you forwarded these messages to	82	28.9
Did any of them told you she implemented the directives in the forwarded preventive methods	22	7.7
Did you bother to ascertain reliability of such information before forwarding the message to others	21	7.3
Do you think that SM can assist to disseminate health information and improve awareness	365	91.3
**Perceived reliability of information from SM (n = 287): multiple responses allowed**		
Always reliable	115	40.1
May be reliable	202	70.4
Usually full of rumours	78	27.2
Usually needs verification	106	36.9
SM can also be used to dispel rumours on health information	332	83
I would like the use of such method of sharing information regulated	337	84.3
Do you think that decisions of others can be influenced through SM use	321	80.3
Government should block sites giving wrong information	341	85.3
Do you think that Government should have used SM in reaching out to youths on EVD outbreak and prevention and control	38	9.5
SM is effective in disseminating health information among youths	206	51.6%

Common misconceptions heard include that it can be treated with antibiotics 51 (17.8%), that it can be prevented by taking daily hot water bath with salt 214(74.6%) and that it can be prevented by taking bitter cola 237(82.6%). One hundred and fifty (52.3%) of them forwarded these messages to others, 82(28.9%) got feedback. Though only 21 (7.3%) bothered to ascertain the reliability of such information before use, 365(91.3%) of respondents believed that SM can assist to disseminate health information and improve awareness.

**Figure 1:**
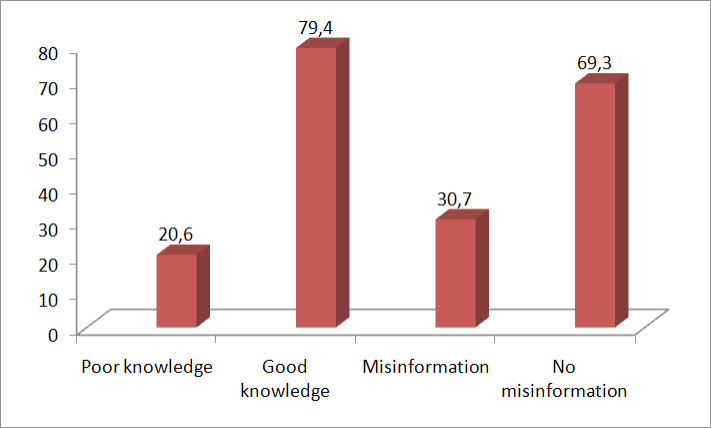
Level of knowledge and misinformation on EVD among respondents using social media

Three hundred and thirty two (83.0%) believed that SM can also be used to dispel rumours on health information, 337(84.3%) said they would like the use of SM regulated, while 341(85.3%) said that government should block sites giving wrong information 206 (51.6%) of respondents still believed that SM is effective in disseminating health information among youths. SM may be useful in this regard. Figure 1 showed that 20.6% had poor while 79.4% had good knowledge of EVD. However 30.7% of respondents had misinformation on EVD while 69.3% got no misinformation on EVD. The good knowledge of EVD information, despite high level of misconception stressed the need for more in-depth information on the subject matter.

Table 3 showed a statistically significantly association exists between level of misconception and gender. Likewise between level of knowledge of EVD and SM being the first source of information on EVD, and believe that SM can assist to disseminate health information and improve awareness (p < 0.05). Further analysis using binary logistic regression showed that male respondents were about one and a half times less likely to have poor knowledge of EVD compared to females (OR 0.6, 95%CI 0.351-1.119, p 0.056), and were also twice more likely to have no associated misconception compared to females (OR 2.1, 95%CI 1.320-3.452, p 0.001). SM being the first source of information were about seven times less likely to have poor level of knowledge (OR 0.14, 95%CI 0.076-0.254, p 0.001), though no difference in level of misconception was found when compared to those who said SM was not their first source of information on EVD (OR 0.9, 95%CI 0.547-1.551, p 0.377).

**Table 3:** Association between knowledge and level of misinformation about EVD and some selected variables

Bi-variate analysis	Knowledge of EVD			Level of misconceptions		
	Poor (n/%)	Good (n/%)	X^2^, p value	Present n/%	None n/%	X^2^, p value
**Sex**						
Male	20(11.6)	153(88.4)	2.809, 2, 0.422	51(29.7)	122(70.3)	1.060, 2, 0.014
Female	39(19.3)	187(82.7)		37(16.4)	189(83.6)	
**SM is the first source of information on EVD**						
Yes	19(6.6)	268(93.4)	5.861, 2, 0.001	62(21.6)	225(78.4)	1.707, 2, 0.426
No	40(37.0)	75(63.0)		26(24.1)	87(75.9)	
SM can assist to disseminate health information and improve awareness						
Yes	50(13.7)	315(86.3)	1.210, 2, 0.002	81(22.2)	234(77.8)	3.829, 2, 0.147
No	7(37.5)	19(62.5)		7(29.2)	17(70.8)	
**Binary logistic regression**	**Knowledge of EVD**			**Level of misconceptions**		
	**95%CI**		**OR, p value**	**95%CI**		**OR, p value**
	**Lower**	**Upper**		**Lower**	**upper**	
Sex (constant = female)	0.351	1.119	0.6, 0.056	1.320	3.452	2.1, 0.001
SM is the first source of information on EVD (constant =no)	0.076	0.254	0.14, 0.001	0.547	1.551	0.9, 0.377
SM can assist to disseminate health information and improve awareness (constant =no)	0.172	1.077	0.4, 0.045	0.336	2.100	0.8, 0.349

Determinants of good level of knowledge about EVD through SM contacts include being a male, having SM as the first source of information on EVD and believing that SM can assist to disseminate and improve health information.

## Discussion

There is an ongoing increase in the use of social media globally [7], including in health care contexts [8]. Majority of students studied were members of one social media or the other. This is the computer technology age, and youths belong to these networks under the guise of peer belonging, socialization, hobby, etc as supported by another study [9]. The backbone of this technology is the GSM handset which is readily accessible and affordable to youths in Nigeria. The characteristics of users of social media for health communication in this study support findings from other studies with 11-34 years being most involved [10, 11]. Some studies reported that there were more female than male users of social network sites in support of this study [12, 13].

The social network media was the first source of information on EVD among many of respondents. This may be so because of the easiness of dissemination of health information generally by the social media at the click of a button, even when the owner of the handset is not there. The numerous misconceptions received by youths could have misled a lot of youths into what to do, as preventive messages were blurred by these wrong information. Since information is being shared on the network, many youths may have shared wrong and unfounded information with their peers and friends. One of such was the information that drinking onions, bitter cola and daily bath with salt in water could treat or prevent EVD. These misconceptions were unfounded. Because information on social network rapidly spread, a large number of youths would have received such information and continue to pass same to others.

Many of the youths studied could not ascertain the origin of information, many forwarded information to others even if not genuine including getting feedbacks through SM Many youths were misled and many took unnecessary and unproven actions. Because of possible misrepresentation, it is always important to ascertain the veracity y of information received through the media generally. Thus, a quality issue has always been a limitation of the use of SM. This include a lack of reliability of the health information [14, 15] and the fact that authors or originators of mails or information or websites are often unidentifiable, or there can be numerous authors, or the line between producer and audience is blurred [16, 17].

Though the ongoing epidemic is the first major outbreak worldwide, several bogous information and misinformation has been spread by non-organized sectors and the communities. News going round about Ebola are frightening, and it has foster suspicion and anxiety in communities and within health care settings and reluctance of people to seek health care or bring their family members for screening and management. The advent of mobile networks which is in tune with the likes of present day youths has assisted in exchange of medically related information. It is therefore important for government who are the custodians of this technology to assume monitoring and evaluation roles in order to regulate this industry, and prevent episodes of mis-information.

The statistically significant association found in this study showed that being a female user of SM is a predisposing factor to processing misinformation on EVD, the class of the respondents or level of education had no significant effect on awareness and getting misinformed. Such relationship between personality traits and engagement with social media has been reported [12, 13]. As stated in this study, the social media remains a veritable means of disseminating information about health and health-related events although bias of perception may result depending on the channel of communication.

Social media provides opportunities for users to generate, share, receive, and comment on social content among multi users through multisensory communication [18, 19]. Thus when in-depth knowledge is lacking, there is a high probability that misinformation and wrong information may be passed from one person to the other- a situation that may also hinder disease control. One major limitation of this study is the paucity of data on EVD awareness and practices, most especially how it relates to the social media. Nearly all available citations are used in this study.

## Conclusion

SM is an important avenue for youths to spread and exchange health information among one another. This medium could have been an avenue to educate the masses and spread messages on EVD prevention and control. As a result of misconceptions and non verification of such information before further processing to peers, the use of SM among youths needs to be regulated. There is a need to create EVD awareness among youths using the SM platform.
